# A Geospatial Analysis of Health, Mental Health, and Stressful Community Contexts in Los Angeles County

**DOI:** 10.5888/pcd16.190138

**Published:** 2019-11-07

**Authors:** Brenda Robles, Courtney S. Thomas, Elaine S. Lai, Tony Kuo

**Affiliations:** 1 Department of Community Health Sciences, UCLA Fielding School of Public Health, Los Angeles, California; 2 Division of Chronic Disease and Injury Prevention, Los Angeles County Department of Public Health, Los Angeles, California; 3 Department of Epidemiology, UCLA Fielding School of Public Health, Los Angeles, California; 4 Department of Family Medicine, David Geffen School of Medicine at UCLA, Los Angeles, California; 5 Population Health Program, UCLA Clinical and Translational Science Institute, Los Angeles, California

## Abstract

**Introduction:**

Despite numerous federal investments, chronic disease continues to disproportionately affect certain communities across the United States. Understanding the regional distribution (including any overlaps) of factors that extend beyond built and food environments, especially factors that may adversely affect chronic disease–related behaviors, is important. This case study of Los Angeles County’s geospatial landscape sought to address these gaps in research and practice.

**Methods:**

We examined the distributions and geographic overlaps between economic hardship, psychological distress, soda consumption, and availability of publicly funded mental health facilities in 8 Service Planning Areas in Los Angeles County. We categorized the geospatial presence of each variable as low, intermediate, or high. We imported all data, collected during 2014–2018, into ArcGIS Pro version 2.3.3 to create 5 bivariate choropleth maps.

**Results:**

Levels of economic hardship were not equally distributed across communities; the county was characterized by intermediate levels of soda consumption and psychological distress. Most areas had low or intermediate availability of publicly funded mental health facilities. We also found some discordance between psychological distress and availability of publicly funded mental health facilities, and between economic hardship and availability of these facilities.

**Conclusion:**

The need exists to address disparities in economic hardship and to increase access to publicly funded mental health supports and providers in Los Angeles County. The information collected in this case study has policy implications for health, public health, and mental health services planning at the local level.

**Summary**

**What is already known on this topic?**

Chronic disease–related health disparities are attributed to factors that extend beyond improvements to the built and food environments, yet few studies have analyzed the geospatial distributions of and connections between factors that have the potential to accentuate or attenuate chronic disease risk.

**What is added by this report?**

Bivariate maps depict the overlap between chronic disease–related psychosocial and behavioral risk factors.

**What are the implications for public health practice?**

Such a geospatial landscape analysis can illuminate areas of high need and how best to prioritize and disseminate scarce resources to advance public health.

## Introduction

Reducing the burden of chronic disease at the population level requires consideration of factors that extend beyond improvements to the built and food environments. This observation is based on a growing body of evidence that points to the importance of psychosocial community dynamics in shaping a person’s decisions about health (1,2). For example, people exposed to chronically stressful community environments may cope with their stress by engaging in unhealthy behaviors, thereby triggering the release of dopamine or similar “feel good” chemicals in the brain (1). Although these behaviors may temporarily reduce feelings of psychological distress, they cumulatively contribute to a greater risk of developing chronic disease (1).

Sugar-sweetened beverage (SSB) consumption is an example of a risk behavior that can provide relief during stressful situations. Studies have shown that SSBs are addictive primarily because of their high fructose content (3,4). Fructose weakens leptin signaling in the brain, thereby increasing hunger and the desire to overconsume food or beverages (3–5). Because SSBs are also easily accessible and low cost (6), curbing this risk behavior is challenging (7), despite millions of US dollars being spent annually to address this problem (8–10). This unhealthy behavior can be more pronounced under high-stress conditions, where mental health supports (eg, counseling, anxiety/depression treatment services) are often inadequate. These and other data suggest that stressful community contexts can affect psychological well-being, which in turn can shape chronic disease risk.

The objective of this case study was to describe the geospatial distributions and connectivity of psychosocial and behavioral factors that can accentuate or attenuate chronic disease risk in Los Angeles County. The case study is among the first in the literature to provide such a snapshot of a large urban jurisdiction.

## Methods

**Figure 1:**
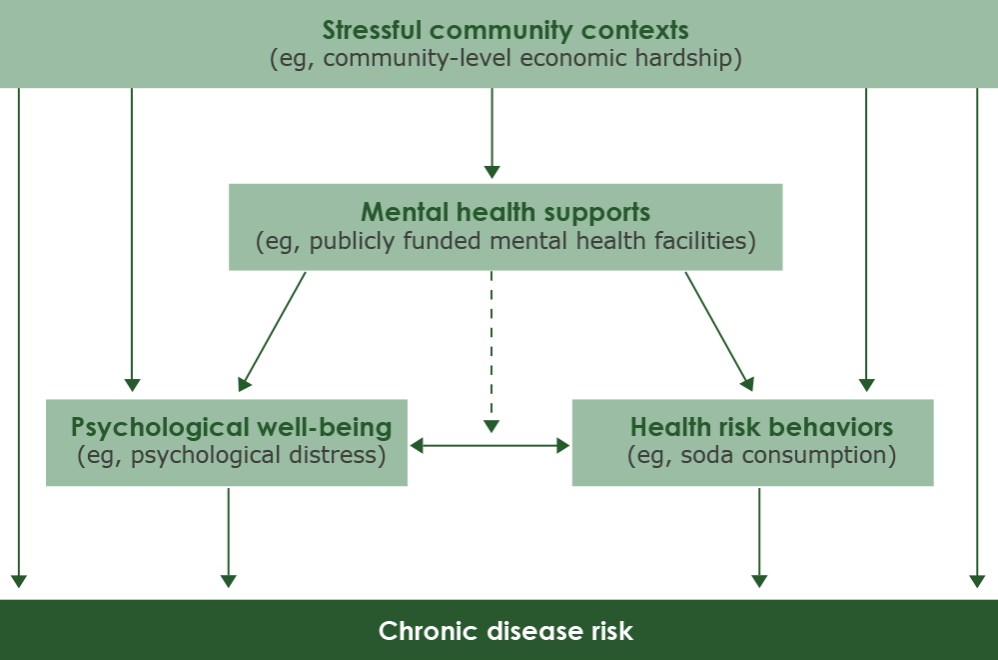
Conceptual model of possible relationships among factors that can influence chronic disease risk.

We used a conceptual model to guide our ecologic descriptive analysis (Figure 1). Los Angeles County is an ideal site for this urban case study because it is one of the most populous (>10 million people) and racially/ethnically diverse counties in the nation (11). The primary unit of analysis was the Service Planning Area (SPA), which is commonly used for program planning purposes in Los Angeles County (12). Each of the 8 SPAs has a unique racial/ethnic distribution, and the prevalence of obesity, overweight, and depression among adults varies by SPA, as does the percentage of adults who reported seeking mental health care in the previous year (Table 1). All data were collected from November 2014 through April 2018.

**Table 1:** Overview of Service Planning Areas (SPAs), Communities Served, and Population Characteristics in Los Angeles County, 2014–2018

SPA and Communities Served^a^	Percentage of Adults^b^					
	Racial/Ethnic Distribution	Obese	Overweight	Current Depression	At Risk for Major Depression	Reported Seeking Mental Health Care in the Last Year
**SPA 1: Antelope Valley**						
Acton, Agua Dulce, Gorman, Lake Hughes, Lake Los Angeles, Lancaster, Littlerock, Palmdale, Quartz Hill, and others	Latino, 44.8%; white, 34.6%; African American, 16.2%; Asian, 3.8%; Native Hawaiian or Other Pacific Islander, 0.2%; American Indian/Alaska Native, 0.4%	29.6	37.0	12.5	13.4	10.1
**SPA 2: San Fernando Valley**						
Burbank, Calabasas, Canoga Park, Canyon Country, Encino, Glendale, La Cañada-Flintridge, San Fernando, Sherman Oaks, Sun Valley, Van Nuys, Woodland Hills, and others	Latino, 40.2%; white, 44.6%; African American, 3.5%; Asian, 11.5%; Native Hawaiian or Other Pacific Islander, 0.1%; American Indian/Alaska Native, 0.2%	19.8	37.0	8.0	10.1	7.0
**SPA 3: San Gabriel Valley**						
Alhambra, Altadena, Arcadia, Azusa, Baldwin Park, Claremont, Covina, Diamond Bar, Duarte, El Monte, Glendora, Irwindale, Monrovia, Monterey Park, Pasadena, Pomona, San Dimas, San Gabriel, San Marino, Temple City, Walnut, West Covina, and others	Latino, 46.3%; white, 21.2%; African American, 3.7%; Asian, 28.6%; Native Hawaiian or Other Pacific Islander, 0.1%; American Indian/Alaska Native, 0.2%	23.8	35.0	6.4	11.0	5.4
**SPA 4: Metro Los Angeles**						
Boyle Heights, Central City, Downtown LA, Echo Park, El Sereno, Hollywood, Mid-City Wilshire, Monterey Hills, Mount Washington, Silverlake, West Hollywood, and Westlake	Latino, 51.8%; white, 24.8%; African American, 5.2%; Asian, 17.9%; Native Hawaiian or Other Pacific Islander, 0.1%; American Indian/Alaska Native, 0.2%	22.1	34.4	10.8	15.7	12.3
**SPA 5: West**						
Beverly Hills, Brentwood, Culver City, Malibu, Pacific Palisades, Playa del Rey, Santa Monica, and Venice	Latino, 16.0%; white, 64.0%; African American, 5.7%; Asian, 14.0%; Native Hawaiian or Other Pacific Islander, 0.1%; American Indian/Alaska Native, 0.2%	10.3	31.1	11.1	6.8	14.2
**SPA 6: South**						
Athens, Compton, Crenshaw, Florence, Hyde Park, Lynwood, Paramount, and Watts	Latino, 68.2%; white, 2.4%; African American, 27.4%; Asian, 1.7%; Native Hawaiian or Other Pacific Islander, 0.2%; American Indian/Alaska Native, 0.1%	34.1	33.4	8.4	16.8	8.1
**SPA 7: East**						
Artesia, Bell, Bellflower, Bell Gardens, Cerritos, City of Commerce, City Terrace, Cudahy, Downey, East Los Angeles, Hawaiian Gardens, Huntington Park, La Habra Heights, Lakewood, La Mirada, Los Nietos, Maywood, Montebello, Norwalk, Pico Rivera, Santa Fe Springs, Signal Hill, South Gate, Vernon, Walnut Park, Whittier, and others	Latino, 73.5%; white, 14.0%; African American, 3.0%; Asian, 9.0%; Native Hawaiian or Other Pacific Islander, 0.2%; American Indian/Alaska Native, 0.2%	28.0	39.1	8.3	11.7	7.9
**SPA 8: South Bay**						
Athens, Avalon, Carson, Catalina Island, El Segundo, Gardena, Harbor City, Hawthorne, Inglewood, Lawndale, Lennox, Long Beach, Hermosa Beach, Manhattan Beach, Palos Verdes Estates, Rancho Dominguez, Rancho Palos Verdes, Redondo Beach, Rolling Hills, Rolling Hills Estates, San Pedro, Wilmington, and others	Latino, 40.4%; white, 28.4%; African American, 14.8%; Asian, 15.4%; Native Hawaiian or Other Pacific Islander, 0.9%; American Indian/Alaska Native, 0.2%	24.1	37.2	12.5	13.4	9.3

a Based on Los Angeles County Department of Public Health Service Planning Area map designations (12).

b Based on the Los Angeles County Department of Public Health report on the key indicators of health (13).

All study protocols and materials were reviewed and approved by the Los Angeles County Department of Public Health Institutional Review Board before data collection. The case study was considered exempt by the institutional review boards at the University of California, Los Angeles, because data for analyses had already been collected from human participants during previous projects.

Los Angeles County is home to several low-income communities that have historically had and continue to have a high prevalence of chronic diseases (13); these diseases are associated with high health care costs (14). In the past decade, the county has been the site of federally funded prevention efforts that targeted obesity, diabetes, heart disease, and stroke (10). During that same period, many people in the Los Angeles County population had poor mental health and inadequate access to mental health supports (eg, behavioral health services) (15,16). Structural and service deficiencies may have contributed to or exacerbated the high prevalence of physical illness in the region (15,16). The constellation of health and mental health problems resulted in recent calls to action that tasked local health authorities to better coordinate the delivery of care across programs that address physical health, mental health, substance abuse, and other social services (17).

### Study population

The study population was recruited from the 2014 Los Angeles County Injury and Violence Prevention Survey (IVPS) (Los Angeles County Department of Public Health, unpublished data, 2014). This cross-sectional internet panel survey was conducted by a California firm that specializes in this type of survey. IVPS was a 1-time assessment commissioned by the Los Angeles County Department of Public Health. Subscribers associated with the panel were first asked to complete a questionnaire that the firm used as part of its standard protocol to screen for eligibility. Those who met the IVPS eligibility criteria (aged ≥18 y and a resident of Los Angeles County) were invited to participate. To recruit a sample that aligned closely with the county’s 2010 US Census demographics (18), the firm applied quota targets in enrollment procedures by sampling until recruitment saturation was met for each sociodemographic stratum. We compared selected characteristics of IVPS respondents with characteristics of the 2010 US Census quota targets for adults (Table 2). Of the 22,397 subscribers who were initially invited to participate, 3,020 clicked into the survey, of whom 1,421 were excluded because they did not meet survey qualifiers (ie, were aged <18 y and not a resident of Los Angeles County) or because of over quotas (ie, the number of respondents in each sociodemographic quota stratum had exceeded the 2010 US Census targets). Of the remaining 1,599 eligible subscribers who started the survey, 1,000 completed it.

**Table 2:** Characteristics of Respondents (N = 1,000) From the 2014 Los Angeles County Injury and Violence Prevention Survey (IVPS) and County Population Estimates From the 2010 US Census^a^

Characteristic	2014 IVPS, %	2010 US Census for Los Angeles County, %
**Sex**		
Female	49.1	48.7
Male	50.9	51.3
**Age, y**		
18–29	38.4	24.6
30–44	36.2	28.9
45–54	11.3	18.5
55–64	9.0	13.7
≥65	5.1	14.4
**Race/ethnicity**		
Hispanic/Latino	48.1	48.1
Black	9.0	6.7
White	26.6	27.6
Asian/Pacific Islander	15.0	14.2
Other	1.3	3.5
**Income, $**		
<25,000	22.2	22.5
25,000–49,999	23.3	22.9
50,000–74,999	18.1	17.6
75,000–99,999	12.6	12.0
100,000–149,999	12.7	13.4
≥150,000	11.1	11.5
**Education**		
High school or less	19.0	44.6
Some college^b^	35.9	29.1
College^c^	45.0	26.3

a Data sources: Los Angeles County Injury and Violence Prevention Survey (N = 1,000) (Los Angeles County Department of Public Health, unpublished data, 2014) and US Census Bureau (18). Some of the variables or categories used in the 2014 IVPS internet panel survey were grouped together to align with some of the variables or categories used in the 2010 US Census. Population estimates from the Census formed the quota criteria that were used in the IVPS survey. Percentages in each category may not sum to 100 because of rounding.

b In the 2014 IVPS internet panel survey, “some college” corresponds to the responses of “technical/vocational school” or “some college.”

c In the 2014 IVPS internet panel survey, “college” corresponds to the responses of “college graduate” or “post-graduate.”

The IVPS was administered in English via the firm’s web-based survey platform from October 10 to November 15, 2014. Questions were closed-ended, with respondents asked to choose their answers from a short list of possible response options or by providing a numeric answer. The final participation rate in the survey was approximately 33% (1,000 of 3,020).

### Variables

**Community-level economic hardship.** We mapped community-level economic hardship, an indicator of stressful community contexts, by using data from the 2008–2012 Los Angeles County Economic Hardship Index, which were linked to survey respondents’ zip codes. The index was constructed by the Los Angeles County Department of Public Health and is a composite score of 6 social and economic indicators: crowded housing, poverty, unemployment, education, dependency (percentage of population aged <18 y or >64 y), and per capita income. It aligns with a previously created composite score created by the Nelson A. Rockefeller Institute of Government (19). The development and application of the index is described elsewhere (20). The Economic Hardship Index used in our study corresponds to 121 places (eg, municipality, town, postal place) and Los Angeles city council districts; the scores are based on 5‐year estimates from the American Community Survey (2008–2012). Economic Hardship Index scores, which ranged in our study from 13.2 (lowest level of hardship) to 82.5 (highest level of hardship), were categorized into tertiles by using the minimum and maximum average score in each zip code. The 3 categories were low (score, 13.2–27.5), intermediate (score, 27.6–55.0), and high (score, 55.1–82.5). These categories were used in a previous study (21).

**Psychological distress.** Psychological distress, an indicator of psychological well-being, was measured by using IVPS respondents’ responses to the 5-item Mental Health Inventory (MHI-5). The MHI-5 asked respondents to report (in the last month) their level of happiness, level of calm and peace, level of nervousness, level of feeling “downhearted and blue,” and level of feeling “so down in the dumps” that nothing could cheer them up (22). Respondents chose from 6 possible response options that assigned scores ranging from 5 to 30 points each. Responses were 5 (none of the time), 10 (a little bit of the time), 15 (some of the time), 20 (a good bit of the time), 25 (most of the time) and 30 (all of the time). We linearly transformed the responses, yielding total scores that ranged from 0 to 100 across zip codes, with higher scores indicating higher levels of psychological distress. We grouped scores into 3 categories: low (score, 0-40), intermediate (score, 41–72), or high (score, ≥73). Previous studies used similar cutoffs (23,24).

**Soda consumption.** Soda consumption, a proxy indicator of health risk behaviors, was also measured by using data from the IVPS data set. This variable was based on a question that asked respondents, “In an average week, about how many regular sodas such as Coke or Mountain Dew, do you drink? Do not include diet sodas or sugar-free drinks. Please count a 12-ounce can, bottle or glass as one drink.” Responses were reported in whole numbers; we grouped responses as low (0 sodas per week), intermediate (1–6 sodas per week), or high (≥7 sodas per week). The cutoffs were the same as in a previous analysis conducted in Los Angeles County (25).

**Availability of publicly funded mental health facilities.** Availability of these facilities, an indicator of mental health supports, was measured using data from the 2018 Los Angeles County Department of Mental Health Providers Locations data set, which was downloaded as a shapefile from the Los Angeles County GIS Data Portal (26). This geospatial dataset contained information on the availability of publicly funded mental health facilities in Los Angeles County, including the name and address of the facility, the SPA and supervisorial district in which the facility was located, the languages and cultures of the people served, and a description of the types of publicly funded program services provided at inpatient, outpatient, and residential publicly funded mental health service locations. We linked these data to the zip codes of IVPS respondents. Then we determined the number of mental health facilities per zip code by using the minimum and maximum number of facilities in each zip code. We categorized availability as low (0–0.9 facilities per zip code), intermediate (1.0–5.9 facilities per zip code), or high (6.0–10.0 facilities per zip code). The cutoffs were based on the histogram distribution of the observed data.

### Analysis

We created a master tabular database in Stata version 14.1 (StataCorp LLC). The database was imported and merged into Esri ArcGIS Pro version 2.3.3. We used the latter software to create 5 bivariate choropleth maps that depicted frequencies of survey responses to each variable of interest, by zip code and by SPA. The maps compared the geographic distributions between 1) economic hardship and psychological distress, 2) economic hardship and soda consumption, 3) psychological distress and soda consumption, 4) psychological distress and availability of publicly funded mental health facilities, and 5) economic hardship and publicly funded mental health facilities.

In the bivariate mapping, we created a coding scheme that assigned a number to each combination of variables. For example, in the comparison of community-level economic hardship and soda consumption, if the score for community-level economic hardship was categorized as low and the score for soda consumption was categorized low, we assigned the number 1, and so forth. This categorization approach was informed by guidance on bivariate choropleth mapping (27). To each of 9 possible combinations of numbers, we assigned a color based on recommendations for selecting map color schemes (28). We overlaid the boundaries of the 8 SPAs onto each map.

We performed additional subanalyses in Stata 14.1. The first subanalysis used the Kruskal–Wallis test to examine differences in sociodemographic and other characteristics among respondents by SPA. We hypothesized that respondent characteristics would vary across the 8 SPAs. The second subanalysis tested the assumption that the relationships indicated by the 5 bivariate maps would vary. We used Pearson χ**^2^** tests to assess differences among the various combinations of map variables categorized as low, intermediate, and high.

## Results

**Figure 2:**
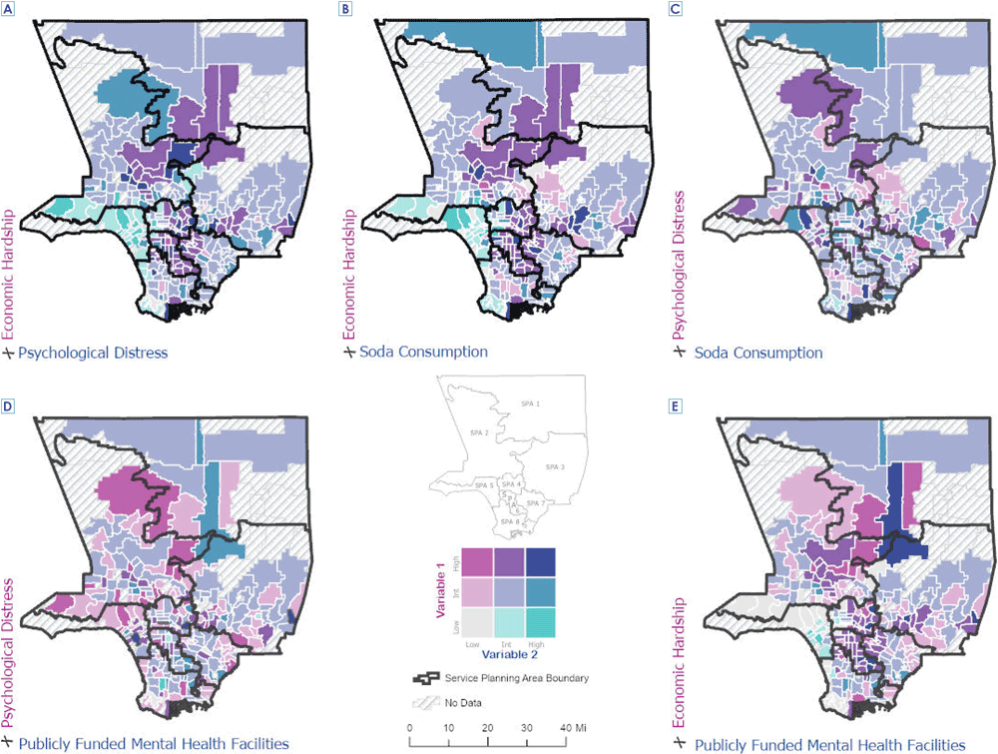
Geospatial comparison of community-level economic hardship, soda consumption, psychological distress, and availability of publicly funded mental health facilities in Los Angeles County, 2014–2018. Abbreviation: Int, intermediate.

We observed several geospatial relationships in the bivariate choropleth maps (Figure 2). The map depicting the relationship between economic hardship and psychological distress (Figure 2A) shows that levels of economic hardship were lowest among the coastal areas of Los Angeles County, particularly in SPA 5, and concentrated in SPA 6 and in pockets in SPA 4, SPA 3, SPA 2, and SPA 1. Most variables for psychological distress were characterized as intermediate. Discordance between levels of economic hardship and psychological distress (eg, low levels of economic hardship and high levels of psychological stress) was more observable across SPAs than within SPAs. Surprisingly, areas depicted as having high levels of psychological distress were also depicted as having low levels of economic hardship — SPA 5 and parts of SPA 2 and SPA 8**.** Conversely, most of SPA 6 and parts of SPA 4, SPA 3, SPA 2, and SPA 1 — areas corresponding to the highest levels of hardship— had intermediate levels of psychological distress.

The map showing the relationship between economic hardship and soda consumption is characterized by intermediate levels of soda consumption, regardless of levels of economic hardship (Figure 2B). Only a few areas in and across the 8 SPAs had low levels or high levels of soda consumption. Soda consumption was most concentrated in the coastal areas, with discordance in and across SPAs. We also found discordance between psychological distress and soda consumption across SPAs (Figure 2C).

Overall, few areas in Los Angeles County had high levels of publicly funded mental health facilities. We found some discordance between psychological distress and availability of publicly funded mental health facilities (Figure 2D) and between economic hardship and availability of facilities (Figure 2E).

With the exception of sex, we found significant differences in characteristics among respondents in the 8 SPAs (Table 3). We also found significant differences in the relationships depicted in the mapping analyses (Table 4). All relationships were significant, except for those between psychological distress and availability of publicly funded mental health facilities.

**Table 3:** Characteristics of Respondents From the 2014 Los Angeles County Injury and Violence Prevention Survey (IVPS) by Service Planning Area (SPA)^a^

Characteristic	All	SPA 1	SPA 2	SPA 3	SPA 4	SPA 5	SPA 6	SPA 7	SPA 8	*P* Value^b^
**% (No.)**	100 (1,000)	4.1 (41)	15.3 (153)	14.8 (148)	18.7 (187)	8.1 (81)	13.9 (139)	9.8 (98)	15.3 (153)	—
**Sociodemographic Characteristics**										
**Sex**										
Female	49.1 (491)	41.5 (17)	41.2 (63)	55.4 (82)	43.9 (82)	50.6 (41)	50.4 (70)	59.2 (58)	51.0 (78)	.06
Male	50.9 (509)	58.5 (24)	58.8 (90)	44.6 (66)	56.2 (105)	49.4 (40)	49.6 (69)	40.8 (40)	49.0 (75)	
**Age, y**										
18–29	38.4 (384)	26.8 (11)	41.2 (63)	31.8 (47)	39.6 (74)	23.5 (19)	54.0 (75)	39.8 (39)	36.6 (56)	<.001
30–44	36.2 (362)	39.0 (16)	25.5 (39)	36.5 (54)	46.0 (86)	50.6 (41)	31.7 (44)	34.7 (34)	31.4 (48)	
45–54	11.3 (113)	12.2 (5)	15.7 (24)	11.5 (17)	8.6 (16)	9.9 (8)	5.0 (7)	13.3 (13)	15.0 (23)	
55–64	9.0 (90)	14.6 (6)	10.5 (16)	10.8 (16)	4.8 (9)	8.6 (7)	7.2 (10)	6.1 (6)	13.1 (20)	
≥65	5.1 (51)	7.3 (3)	7.2 (11)	9.5 (14)	1.1 (2)	7.4 (6)	2.2 (3)	6.1 (6)	3.9 (6)	
**Race/ethnicity**										
Hispanic/Latino	48.1 (481)	48.8 (20)	33.3 (51)	43.9 (65)	62.0 (116)	29.6 (24)	57.6 (80)	74.5 (73)	34.0 (52)	<.001
Non-Hispanic black	9.0 (90)	9.8 (4)	3.3 (5)	4.7 (7)	5.4 (10)	6.2 (5)	20.1 (28)	4.1 (4)	17.7 (27)	
Non-Hispanic white	26.6 (266)	36.6 (15)	49.0 (75)	20.3 (30)	21.9 (41)	42.0 (34)	14.4 (20)	17.4 (17)	22.2 (34)	
Asian/Pacific Islander	15.0 (150)	4.9 (2)	12.4 (19)	29.7 (44)	10.7 (20)	21.0 (17)	5.8 (8)	2.0 (2)	24.8 (38)	
Other	1.3 (13)	0	2.0 (3)	1.4 (2)	0	1.2 (1)	2.2 (3)	2.0 (2)	1.3 (2)	
**Income, $**										
<25,000	22.2 (222)	29.3 (12)	23.5 (36)	24.3 (36)	17.7 (33)	9.9 (8)	25.2 (35)	25.5 (25)	24.2 (37)	<.001
25,000–49,999	23.3 (233)	31.7 (13)	22.2 (34)	21.0 (31)	24.1 (45)	13.6 (11)	24.5 (34)	30.6 (30)	22.9 (35)	
50,000–74,999	18.1 (181)	9.8 (4)	15.7 (24)	23.7 (35)	13.4 (25)	22.2 (18)	20.1 (28)	16.3 (16)	20.3 (31)	
75,000–99,999	12.6 (126)	7.3 (3)	9.8 (15)	11.5 (17)	20.3 (38)	18.5 (15)	11.5 (16)	9.2 (9)	8.5 (13)	
100,000–149,999	12.7 (127)	7.3 (3)	13.7 (21)	10.1 (15)	17.1 (32)	16.1 (13)	7.9 (11)	12.2 (12)	13.1 (20)	
≥150,000	11.1 (111)	14.6 (6)	15.0 (23)	9.5 (14)	7.5 (14)	19.8 (16)	10.8 (15)	6.1 (6)	11.1 (17)	
**Education**										
High school or less	19.0 (190)	26.8 (11)	13.7 (21)	25.0 (37)	14.4 (27)	8.6 (7)	24.5 (34)	22.5 (22)	20.3 (31)	<.001
Some college^c^	35.8 (258)	41.5 (17)	36.0 (55)	38.5 (57)	31.6 (59)	19.8 (16)	36.7 (51)	49.0 (48)	36.0 (55)	
College^d^	45.0 (450)	31.7 (13)	50.3 (77)	35.8 (53)	54.0 (101)	71.6 (58)	38.9 (54)	28.6 (28)	43.1 (66)	
**Stressful Community Contexts**										
**Community-level economic hardship^e^**										
Low	26.9 (269)	9.8 (4)	24.2 (37)	18.2 (27)	40.1 (75)	98.8 (80)	6.5 (9)	2.0 (2)	22.9 (35)	<.001
Intermediate	40.5 (405)	90.2 (37)	48.4 (74)	58.8 (87)	19.3 (36)	1.2 (1)	5.0 (7)	63.3 (62)	66.0 (101)	
High	32.6 (326)	0	27.5 (42)	23.0 (34)	40.6 (76)	0	88.5 (123)	34.7 (34)	11.1 (17)	
**Mental Health Supports**										
**Availability of publicly funded mental health facilities^f^**										
Low	29.9 (299)	46.3 (19)	39.9 (61)	31.1 (46)	20.9 (39)	50.6 (41)	7.9 (11)	23.5 (23)	38.6 (59)	<.001
Intermediate	58.2 (582)	29.3 (12)	51.6 (79)	57.4 (85)	62.0 (116)	32.1 (26)	81.3 (113)	65.3 (64)	56.9 (87)	
High	9.3 (93)	24.4 (10)	2.0 (3)	10.1 (15)	17.1 (32)	16.1 (13)	9.4 (13)	3.1 (3)	2.6 (4)	
**Psychological Well-Being**										
**Psychological distress^g^**										
Low	44.3 (443)	68.3 (28)	44.4 (68)	46.6 (69)	39.0 (73)	38.3 (31)	35.3 (49)	51.0 (50)	49.0 (75)	.007
Intermediate	48.9 (489)	29.3 (12)	49.7 (76)	46.6 (69)	54.6 (102)	55.6 (45)	56.1 (78)	43.9 (43)	41.8 (64)	
High	6.8 (68)	2.4 (1)	5.9 (9)	6.8 (10)	6.4 (12)	6.2 (5)	8.6 (12)	5.1 (5)	9.2 (14)	
**Health Risk Behaviors**										
**Soda consumption^h^**										
Low	23.9 (239)	24.4 (10)	34.0 (52)	22.3 (33)	18.7 (35)	39.5 (32)	18.0 (25)	17.4 (17)	22.9 (35)	.03
Intermediate	55.6 (556)	53.7 (22)	49.0 (75)	58.8 (87)	59.4 (111)	37.0 (30)	64.0 (89)	55.1 (54)	57.5 (88)	
High	20.4 (204)	22.0 (9)	17.0 (26)	18.9 (28)	21.9 (41)	23.5 (19)	17.3 (24)	27.6 (27)	19.6 (30)	

a Data source: Los Angeles County Injury and Violence Prevention Survey (N = 1,000) ((Los Angeles County Department of Public Health, unpublished data, 2014). Numbers or percentages in each column may not add up to the total or to 100% because of missing information or rounding.

b *P* values determined by Kruskall–Wallis test.

c In the 2014 IVPS internet panel survey, “some college” corresponds to the responses of “technical/vocational school” or “some college.”

d In the 2014 IVPS internet panel survey, “college” corresponds to the responses of “college graduate” or “post-graduate.”

e Measured by the 2008–2012 Los Angeles County Economic Hardship Index, a composite score of 6 social and economic indicators: crowded housing, poverty, unemployment, education, dependency (percentage of population aged <18 y or >64 y), and per capita income (19,20). Scores ranged from 13.2 (lowest level of hardship) to 82.5 (highest level of hardship) and were categorized into tertiles: low (score, 13.2–27.5), intermediate (score, 27.6–55.0), and high (score, 55.1–82.5).

f Data from the 2018 Los Angeles County Department of Mental Health Providers Locations data set, downloaded as a shapefile from the Los Angeles County GIS Data Portal (26). We categorized availability as low (0–0.9 facilities per zip code), intermediate (1.0–5.9 facilities per zip code), and high (6.0-10.0 facilities per zip code).

g Measured by using IVPS respondents’ responses to the 5-item Mental Health Inventory (22). Higher scores indicate higher levels of distress. Scores, ranging from 0 to 100, were grouped into 3 categories: low (score, 0–40), intermediate (score, 41–72), and high (score, ≥73).

h Measured by using data from the IVPS data set. Question asked, “In an average week, about how many regular sodas such as Coke or Mountain Dew, do you drink? Do not include diet sodas or sugar-free drinks. Please count a 12-ounce can, bottle or glass as one drink.” Responses were categorized as low (0 sodas), intermediate (1–6 sodas), and high (≥7 sodas).

**Table 4:** Subanalyses of the Relationships Between Community-Level Economic Hardship, Psychological Distress, Soda Consumption, and Availability of Publicly Funded Mental Health Facilities, Los Angeles County, 2014–2018^a^

Characteristic	Low, No. (%)	Intermediate, No. (%)	High, No. (%)	*P* Value^b^
**Community-Level Economic Hardship^c^**				
**Psychological distress^d^**				
Low	107 (39.8)	207 (51.1)	129 (39.6)	.004
Intermediate	145 (53.9)	177 (43.7)	167 (51.2)	
High	17 (6.3)	21 (5.2)	30 (9.2)	
**Soda consumption^e^**				
Low	89 (33.1)	92 (22.7)	58 (17.8)	.001
Intermediate	126 (46.8)	237 (58.5)	193 (59.2)	
High	54 (20.1)	75 (18.5)	74 (22.7)	
**Availability of publicly funded mental health facilities^f^**				
Low	134 (49.8)	140 (34.6)	25 (7.7)	<.001
Intermediate	98 (36.4)	236 (59.3)	248 (76.1)	
High	28 (10.4)	18 (4.4)	47 (14.4)	
**Psychological Distress^d^**				
**Soda consumption^e^**				
Low	128 (28.9)	88 (18.0)	23 (33.8)	.002
Intermediate	234 (52.8)	288 (58.9)	34 (50.0)	
High	81 (18.3)	111 (22.7)	11 (16.2)	
**Availability of publicly funded mental health facilities^f^**				
Low	141 (31.8)	141 (28.8)	17 (25.0)	.07
Intermediate	260 (58.7)	278 (56.9)	44 (64.7)	
High	36 (8.1)	50 (10.2)	7 (10.3)	

a Data source: Los Angeles County Injury and Violence Prevention Survey (IVPS) (N = 1,000) (Los Angeles County Department of Public Health, unpublished data, 2014). Numbers or percentages in each column may not add up to the total or to 100% because of missing information or rounding.

b Pearson χ^2^ test.

c Measured by 2008–2012 Los Angeles County Economic Hardship Index, a composite score of 6 social and economic indicators: crowded housing, poverty, unemployment, education, dependency (percentage of population aged <18 y or >64 y), and per capita income (19,20). Scores ranged from 13.2 (lowest level of hardship) to 82.5 (highest level of hardship) and were categorized into tertiles: low (score, 13.2–27.5), intermediate (score, 27.6–55.0), and high (score, 55.1–82.5).

d Measured by using IVPS respondents’ responses to the 5-item Mental Health Inventory (22). Higher scores indicate higher levels of distress. Scores, ranging from 0 to 100, were grouped into 3 categories: low (score, 0–40), intermediate (score, 41–72), and high (score, ≥73).

e Measured by using data from the IVPS data set. Question asked, “In an average week, about how many regular sodas such as Coke or Mountain Dew, do you drink? Do not include diet sodas or sugar-free drinks. Please count a 12-ounce can, bottle or glass as one drink.” Responses were categorized as low (0 sodas), intermediate (1–6 sodas), and high (≥7 sodas).

f Data from the 2018 Los Angeles County Department of Mental Health Providers Locations data set, downloaded as a shapefile from the Los Angeles County GIS Data Portal (26). We categorized availability as low (0–0.9 facilities per zip code), intermediate (1.0–5.9 facilities per zip code), and high (6.0–10.0 facilities per zip code).

## Discussion

We note 4 chief findings in our case study. First, levels of economic hardship were not equally distributed across the population in Los Angeles County. For example, SPA 6 had the highest hardship levels. Its population is predominantly Hispanic (68.5%) and African American (27.8%); more than a third (34%) of the population has a household income of less than 100% of the federal poverty level (13). In contrast, coastal areas, such as those in SPA 5, had populations that were predominantly white (64.0%) and Asian (14.0%), with only about 12% having a household income of less than 100% of the federal poverty level (13). Disparities by social and economic statuses across racial/ethnic lines have been previously described (29,30).

Second, our case study characterized levels of soda consumption as intermediate or high, regardless of economic hardship. Soda consumption was highest in areas with low or intermediate levels of economic hardship. This finding suggests that soda consumption may be influenced by the low cost and ubiquity of SSBs in these food environments. Given that this health behavior has multifactorial and complex roots and cannot be viewed simply along socioeconomic lines, public health messaging and resources may be needed to educate residents about these SSBs and about ways to reduce their consumption.

Third, our geospatial results suggest that although levels of psychological distress were high among Los Angeles County residents, a shortage of publicly funded mental health facilities (providers) exists in the region. This finding underscores the need to augment delivery of behavioral health services in Los Angeles County, such as those that are currently being planned (31). Mapping the availability of mental health supports in relation to other community contexts is timely and can strengthen integrated delivery of health services and behavioral health services.

Finally, the bivariate maps illustrated a discordance in geographic distributions of stressful community contexts, psychological well-being, health risk behaviors, and availability of mental health supports in and/or across SPAs. This finding highlights a need for program planners to better tailor chronic disease prevention that emphasizes meeting the needs of target populations.

Our case study has limitations. First, some variables in the mapping analyses came from a cross-sectional data set that may have limited generalizability outside of the target population(s). We took steps to mitigate this limitation (eg, by applying US Census-based quota criteria in the IVPS) and to address other potential sources of bias (eg, selection bias). Second, because data were collected at various time points, our analysis may have introduced temporal bias. However, where feasible, we gathered all key data from data sources that collected data during the same timeframe. Third, ascribing characteristics of individual survey respondents in the IVPS to defined zip codes may have introduced interpretive errors because these geographic data do not necessarily represent geographic regions of Los Angeles County. Zip codes, which correspond to service areas of the US Postal Service and typically denote address groups or delivery routes, have some limitations (eg, zip codes may overlap, boundaries may be artificially constructed). Lastly, the mapping techniques did not account for the overlaps between the various spatial boundaries of zip codes and SPAs.

Despite these limitations, our case study offers a “big picture” framework that may help to guide future health, public health, and mental health services planning in Los Angeles County. The maps provide valuable community-level planning data for chronic disease prevention, especially as they relate to nonhealth factors (eg, social conditions such as economic hardship, psychological distress, availability and adequacy of behavioral health resources) that can influence health.

From a program design–redesign perspective, future mapping analyses could capture data on the emerging need for integrated and coordinated care instead of the status quo, in which services are delivered by providers who do not share information, priorities, tools, or processes. Coordination between health services and behavioral health services, including communication among providers, will need to be strengthened if Los Angeles County is to improve its care of the poor and underserved. Structurally, for this change to happen, workforce development will be critical. For example, although recruiting more licensed professionals in health, public health, and/or mental health (eg, therapists, case managers, primary care physicians) may be the initial goal, diversifying the knowledge and skills of these providers to better acquaint them with concepts in chronic disease prevention and management early in their training may be even more important. Facilitating team care environments may be another avenue for building a more integrated infrastructure. This model of practice can enable health professionals to work with and learn from each other in a more meaningful, seamless way, and to reduce costs by avoiding high-end care (eg, specialists or high-level providers delivering services that other, less costly staff members could deliver).
